# Immunotherapy using bispecific T cell engager (BiTE^®^) antibodies: preclinical and clinical experience in acute leukemia

**DOI:** 10.1186/2051-1426-2-S3-P115

**Published:** 2014-11-06

**Authors:** Marion Subklewe, Max Topp, Christina Krupka, Peter Kufer, Roman Kischel, Thomas Köhnke, Patrick Baeuerle, Gerhard Zugmaier, Stanley Frankel, Tapan Maniar, Katie Newhall, Karsten Spiekermann, Gert Riethmueller, Dirk Nagorsen, Wolfgang Hiddemann

**Affiliations:** 1 Department of Internal Medicine III, Klinikum der Universität München, and Clinical Cooperation Group Immunotherapy at the Helmholtz Institute Munich, Germany; 2 Medizinische Klinik und Poliklinik II, Universitätsklinikum Würzburg, Germany; 3 AMGEN Research (Munich) GmbH, Germany; 4 Amgen Rockville, Inc., United States; 5 Amgen Inc., United States; 6 Department of Internal Medicine III, Klinikum der Universität München, and Clinical Cooperation Group Leukemia at the Helmholtz Institute Munich, Germany; 7 Institute for Immunology, Ludwig-Maximilians-University, Germany; 8 Amgen Inc., Germany

## 

BiTE^®^ antibodies are novel recombinant single chain Ig domain constructs that leverage the endogenous cytotoxic potential of polyclonal T cells to target malignant cells by utilizing the specific binding properties of variable domains from two different antibodies. Antibody-based immunotherapy represents a promising strategy in cancer. BiTE^®^ antibodies have demonstrated efficacy in hematologic malignancies, both preclinically and clinically.

Two investigational BiTE antibodies are under development targeting leukemia. The most advanced BiTE^®^ antibody, Blinatumomab, directs cytotoxic T cells to CD19-expressing target cells. Blinatumomab has shown anti-leukemia activity in adult relapsed/refractory (r/r) B-precursor ALL. Its efficacy and toxicity was evaluated in a large confirmatory Phase II study. Patients with Ph-negative r/r ALL (N = 189; refractory; 1st relapse

Given the anti-leukemia activity of single-agent Blinatumomab in a difficult-to-treat population with r/r ALL, another BiTE^®^ antibody targeting CD33, AMG 330, was developed for its suitability as immunotherapy in AML. To simulate the natural setting of target and T cells in AML patients, a long-term culture system was developed that supports the growth of primary AML cells *ex-vivo *for up to 5 weeks. AMG 330 activated and expanded residual autologous T cells within primary AML patient samples and eliminated CD33+ blasts even at very low effector to target ratios. The functional relevance of CD33 expression levels was shown by faster lysis kinetics of CD33^BRIGHT ^versus CD33^DIM ^AML cell lines and primary AML cells in ex-vivo cytotoxicity assays. However, by extending the exposure time to AMG 330, potent anti-leukemic activity was observed in both CD33^BRIGHT ^and CD33^DIM ^cells. AMG 330 treated T cells were shown to up-regulate the activation markers CD25, PD-1, TIM3 and LAG3, which was partially reversible after complete target cell elimination

Clinical experience with Blinatumomab in ALL and ex-vivo activity of AMG 330 in primary AML samples supports further development of BiTE^®^ antibodies for targeted T cell-mediated immunotherapy of patients with malignancies.

